# Bis[4-(dimethyl­amino)­pyridinium] tetra­chloridozincate

**DOI:** 10.1107/S1600536811005010

**Published:** 2011-02-16

**Authors:** Riadh Kefi, Frederic Lefebvre, Matthias Zeller, Cherif Ben Nasr

**Affiliations:** a Laboratoire de Chimie des Matériaux, Faculté des Sciences de Bizerte, 7021 Zarzouna, Tunisia; b Laboratoire C2P2 (Equipe COMS), Ecole Superieure de Chimie Physique, Electronique, Villeurbanne, France; c Youngstown State University, Department of Chemistry, One University Plaza, Youngstown, Ohio 44555-3663, USA

## Abstract

In the title compound, (C_7_H_11_N_2_)_2_[ZnCl_4_], [ZnCl_4_]^2−^ anions and 4-(dimethyl­amino)­pyridinium cations are held together by various inter­molecular inter­actions including Coulombic attraction, hydrogen bonding and π–π stacking inter­actions. Three Cl atoms of the [ZnCl_4_]^2−^ tetra­hedron act as acceptors in N—H⋯Cl hydrogen bonds. The hydrogen bonds, both of which are bifurcated, lead to the formation of a three-dimensional network. Within the network, inter­molecular π–π stacking inter­actions with a centroid–centroid distance of 3.5911 (7) Å arrange the 4-(dimethyl­amino)­pyridinium cations into anti­parallel dimers.

## Related literature

For common applications of organic–inorganic hybrid materials, see: Kobel & Hanack (1986 ▶); Pierpont & Jung (1994 ▶); Huskins & Robson (1990 ▶). For related structures and discussion of geometrical features, see: Albrecht *et al.* (2003 ▶); El Glaoui *et al.* (2008 ▶).
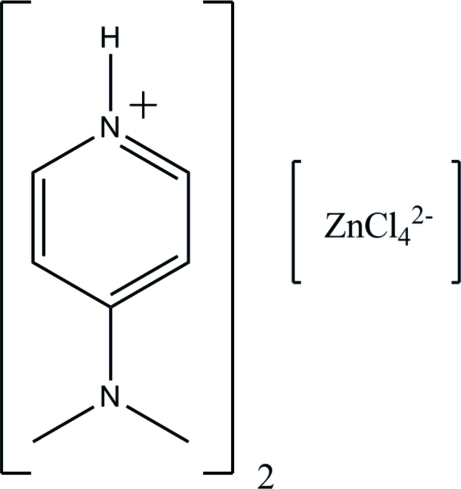


## Experimental

### 

#### Crystal data

(C_7_H_11_N_2_)_2_[ZnCl_4_]
*M* *_r_* = 453.55
Triclinic, 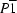

*a* = 7.7056 (8) Å
*b* = 8.2159 (8) Å
*c* = 16.0972 (16) Å
α = 77.422 (1)°
β = 79.804 (1)°
γ = 75.983 (1)°
*V* = 956.67 (17) Å^3^
*Z* = 2
Mo *K*α radiation
μ = 1.85 mm^−1^
*T* = 100 K
0.55 × 0.50 × 0.45 mm

#### Data collection

Bruker SMART APEX CCD diffractometer
Absorption correction: multi-scan (*SADABS*; Bruker, 2009 ▶) *T*_min_ = 0.602, *T*_max_ = 0.746
21487 measured reflections
5803 independent reflections
5638 reflections with *I* > 2σ(*I*)
*R*_int_ = 0.019

#### Refinement

*R*[*F* ^2^ > 2σ(*F* ^2^)] = 0.020
*wR*(*F* ^2^) = 0.053
*S* = 1.07
5803 reflections
212 parameters
H-atom parameters constrained
Δρ_max_ = 0.48 e Å^−3^
Δρ_min_ = −0.44 e Å^−3^

### 

Data collection: *APEX2* (Bruker, 2009 ▶); cell refinement: *SAINT* (Bruker, 2009 ▶); data reduction: *SAINT*; program(s) used to solve structure: *SHELXTL* (Sheldrick, 2008 ▶); program(s) used to refine structure: *SHELXTL*; molecular graphics: *SHELXTL*; software used to prepare material for publication: *SHELXTL*.

## Supplementary Material

Crystal structure: contains datablocks global, I. DOI: 10.1107/S1600536811005010/rk2262sup1.cif

Structure factors: contains datablocks I. DOI: 10.1107/S1600536811005010/rk2262Isup2.hkl

Additional supplementary materials:  crystallographic information; 3D view; checkCIF report

## Figures and Tables

**Table 1:** Hydrogen-bond geometry (Å, °)

*D*—H⋯*A*	*D*—H	H⋯*A*	*D*⋯*A*	*D*—H⋯*A*
N3—H3⋯Cl1^i^	0.88	2.88	3.5090 (11)	130
N3—H3⋯Cl3^i^	0.88	2.46	3.2224 (10)	146
N1—H1*A*⋯Cl2	0.88	2.81	3.4043 (10)	126
N1—H1*A*⋯Cl1	0.88	2.53	3.2066 (10)	134

Symmetry code: (i) 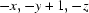.

**Fig. 1.**
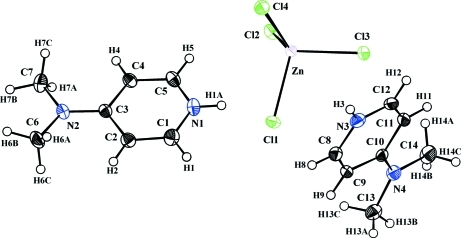
A view of the title compound with the atom numbering scheme. Displacement ellipsoids are drawn at 50% probability level. H atoms are presented as a small spheres of arbitrary radius.

**Fig. 2.**
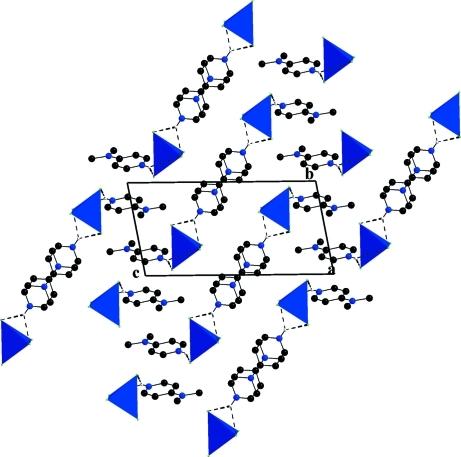
The packing of (C7H11N2)2ZnCl4, viewed down the a axis. Hydrogen bonds are denoted by dotted lines.

## supplementary crystallographic information

### Comment

Organic–inorganic compounds constitute a vast family of hybrid materials of considerable technological importance (Kobel & Hanack, 1986; Pierpont & Jung, 1994; Huskins & Robson, 1990). In this work, we report the molecular and crystal structures of one such compound, (C_7_H_11_N_2_)_2_ZnCl_4_. As shown in Fig. 1, only the nitrogen atom of the aromatic ring of the title compound is protonated, but not the dimethylamine group. Thus, to ensure charge equilibrium, the structure associates each tetrachlorizincate anion with two 4-(dimethylamino)pyridinium cations. Fig.2 shows that the atomic arrangement of the title hybrid material which can be described as inorganic [ZnCl_4_]^2-^ units isolated from each other by the organic cations. The different entities are held together by columbic attraction and multiple hydrogen bonds to form a three dimensional network. Previous studies have shown that the presence of hydrogen bonds influences appreciably the geometrical parameters of the tetrachlorozincate anions (Albrecht *et al.*, 2003). Indeed, the fact that the Zn—Cl4 bond lengh is the shortest meets the expectation derived from hydrogen bonding effects, as the atom Cl4 is not involved in a hydrogen bond, while the remaining Cl1, Cl2 and Cl3 are acting as acceptors of hydrogen bonds with concurrently weakened Zn—Cl bonds. Both of the hydrogen bonds are bifurcated, N1—H1···(Cl1, Cl2) and N3—H3···(Cl1^i^, Cl3^i^) (Table 1). Symmetry code: (i) -*x*, -*y*+1, -*z*. The Cl—Zn—Cl bond angles range from 102.25 (1)° to 117.03 (1)°. These values indicate that the coordination geometry of the zinc atom can be regarded as a slightly distorted tetrahedron, similar as in other related compounds such as 4-(2–ammonioethyl)morpholin–4–ium tetrachlorozincate where the corresponding limit angles are 98.90 (4)° to 114.74 (4)° (El Glaoui *et al.*, 2008). Intermolecular π–π stacking is present between adjacent 4-(dimethylamino)pyridinium cations with a centroid–centroid distance of 3.5911 (7)Å (symmetry code for the second pyridine ring: (i) -*x*, -*y*+1, -*z*).

### Experimental

A mixture of an aqueous solution of 4-(dimethylamino)pyridine (4 mmol, 0.488 g), zinc chloride (2 mmol, 0.396 g) and HCl (10 ml, 0.4 *M*) in a Petri dish was slowly evaporated at room temperature. Crystals of the title compound, which remained stable under normal conditions of temperature and humidity, were isolated after several days and subjected to X–ray diffraction analysis (yield 57%).

### Refinement

Reflection (0 0 1) was obscured by the beamstop and was omitted from the refinement. C—H hydrogen atoms were placed in calculated positions with C—H distances in the range 0.93Å–0.97Å. N—H hydrogen atoms were placed in calculated positions with N—H distances of 0.88Å. The *U*_iso_(H) values of all H atoms were constrained to 1.2(1.5) times *U*_eq_ of the respective parent atom.

### Crystal data

**Table:**

(C_7_H_11_N_2_)_2_[ZnCl_4_]	*Z* = 2
*M**_r_* = 453.55	*F*(000) = 464
Triclinic, *P*1	*D*_x_ = 1.574 Mg m^−^^3^
Hall symbol: -P 1	Mo *K*α radiation, λ = 0.71073 Å
*a* = 7.7056 (8) Å	Cell parameters from 8289 reflections
*b* = 8.2159 (8) Å	θ = 2.6–31.0°
*c* = 16.0972 (16) Å	µ = 1.85 mm^−^^1^
α = 77.422 (1)°	*T* = 100 K
β = 79.804 (1)°	Block, colourless
γ = 75.983 (1)°	0.55 × 0.50 × 0.45 mm
*V* = 956.67 (17) Å^3^	

### Data collection

**Table:**

Bruker SMART APEX CCD diffractometer	5803 independent reflections
Radiation source: fine–focus sealed tube	5638 reflections with *I* > 2σ(*I*)
graphite	*R*_int_ = 0.019
φ and ω scans	θ_max_ = 31.5°, θ_min_ = 2.6°
Absorption correction: multi-scan (*SADABS*; Bruker, 2009)	*h* = −11→11
*T*_min_ = 0.602, *T*_max_ = 0.746	*k* = −11→11
21487 measured reflections	*l* = −23→23

### Refinement

**Table:**

Refinement on *F*^2^	Primary atom site location: structure-invariant direct methods
Least-squares matrix: full	Secondary atom site location: difference Fourier map
*R*[*F*^2^ > 2σ(*F*^2^)] = 0.020	Hydrogen site location: inferred from neighbouring sites
*wR*(*F*^2^) = 0.053	H-atom parameters constrained
*S* = 1.07	*w* = 1/[σ^2^(*F*_o_^2^) + (0.0265*P*)^2^ + 0.3664*P*] where *P* = (*F*_o_^2^ + 2*F*_c_^2^)/3
5803 reflections	(Δ/σ)_max_ = 0.004
212 parameters	Δρ_max_ = 0.48 e Å^−^^3^
0 restraints	Δρ_min_ = −0.44 e Å^−^^3^

### Special details

**Table:**

Geometry. All s.u.'s (except the s.u. in the dihedral angle between two l.s. planes) are estimated using the full covariance matrix. The cell s.u.'s are taken into account individually in the estimation of s.u.'s in distances, angles and torsion angles; correlations between s.u.'s in cell parameters are only used when they are defined by crystal symmetry. An approximate (isotropic) treatment of cell s.u.'s is used for estimating s.u.'s involving l.s. planes.
Refinement. Refinement of *F*^2^ against ALL reflections. The weighted *R*–factor *wR* and goodness of fit *S* are based on *F*^2^, conventional *R*–factors *R* are based on *F*, with *F* set to zero for negative *F*^2^. The threshold expression of *F*^2^ > σ(*F*^2^) is used only for calculating *R*–factors(gt) *etc*. and is not relevant to the choice of reflections for refinement. *R*–factors based on *F*^2^ are statistically about twice as large as those based on *F*, and *R*–factors based on ALL data will be even larger.

### Fractional atomic coordinates and isotropic or equivalent isotropic displacement parameters (Å^2^)

**Table:**

	*x*	*y*	*z*	*U*_iso_*/*U*_eq_	
C1	0.14583 (16)	0.32828 (15)	0.43051 (7)	0.0193 (2)	
H1	0.0707	0.4358	0.4378	0.023*	
C2	0.13978 (15)	0.19127 (14)	0.49549 (7)	0.01699 (19)	
H2	0.0616	0.2037	0.5475	0.020*	
C3	0.25157 (14)	0.02905 (13)	0.48499 (6)	0.01385 (18)	
C4	0.36177 (14)	0.01982 (14)	0.40430 (7)	0.01539 (18)	
H4	0.4353	−0.0863	0.3935	0.018*	
C5	0.36190 (15)	0.16277 (14)	0.34264 (7)	0.01706 (19)	
H5	0.4373	0.1555	0.2895	0.020*	
C6	0.15682 (17)	−0.09487 (17)	0.63337 (7)	0.0221 (2)	
H6A	0.1832	0.0006	0.6528	0.033*	
H6B	0.1952	−0.2011	0.6733	0.033*	
H6C	0.0269	−0.0753	0.6315	0.033*	
C7	0.36088 (17)	−0.27506 (15)	0.53236 (8)	0.0217 (2)	
H7A	0.3255	−0.3030	0.4825	0.033*	
H7B	0.3391	−0.3621	0.5830	0.033*	
H7C	0.4894	−0.2719	0.5215	0.033*	
C8	−0.02081 (15)	0.25984 (14)	−0.12815 (7)	0.01678 (19)	
H8	0.0133	0.2852	−0.1884	0.020*	
C9	0.07683 (14)	0.29557 (13)	−0.07416 (7)	0.01428 (18)	
H9	0.1783	0.3455	−0.0971	0.017*	
C10	0.02760 (14)	0.25842 (13)	0.01653 (6)	0.01374 (18)	
C11	−0.12782 (14)	0.18589 (14)	0.04599 (7)	0.01695 (19)	
H11	−0.1684	0.1612	0.1057	0.020*	
C12	−0.21802 (14)	0.15207 (14)	−0.01175 (8)	0.0186 (2)	
H12	−0.3199	0.1016	0.0085	0.022*	
C13	0.29355 (16)	0.34659 (15)	0.03844 (8)	0.0207 (2)	
H13A	0.3693	0.2723	0.0000	0.031*	
H13B	0.3570	0.3413	0.0869	0.031*	
H13C	0.2679	0.4643	0.0070	0.031*	
C14	0.06780 (18)	0.26180 (17)	0.16320 (7)	0.0250 (2)	
H14A	−0.0625	0.3053	0.1748	0.038*	
H14B	0.1316	0.3219	0.1902	0.038*	
H14C	0.0967	0.1394	0.1868	0.038*	
Cl1	0.21516 (3)	0.72097 (3)	0.315840 (16)	0.01547 (5)	
Cl2	0.55357 (4)	0.45957 (3)	0.189582 (16)	0.01755 (5)	
Cl3	0.49003 (3)	0.93916 (3)	0.147680 (17)	0.01690 (5)	
Cl4	0.72105 (3)	0.67369 (3)	0.331637 (17)	0.01814 (5)	
N1	0.25646 (14)	0.31464 (12)	0.35590 (6)	0.01823 (18)	
H1A	0.2598	0.4057	0.3155	0.022*	
N2	0.25439 (13)	−0.10807 (12)	0.54743 (6)	0.01678 (17)	
N3	−0.16574 (13)	0.18872 (12)	−0.09693 (6)	0.01829 (18)	
H3	−0.2268	0.1661	−0.1328	0.022*	
N4	0.12405 (13)	0.28980 (13)	0.07042 (6)	0.01765 (17)	
Zn1	0.507513 (15)	0.695820 (15)	0.249259 (7)	0.01220 (4)	

### Atomic displacement parameters (Å^2^)

**Table:**

	*U*^11^	*U*^22^	*U*^33^	*U*^12^	*U*^13^	*U*^23^
C1	0.0226 (5)	0.0160 (5)	0.0197 (5)	−0.0029 (4)	−0.0029 (4)	−0.0049 (4)
C2	0.0179 (5)	0.0175 (5)	0.0155 (5)	−0.0035 (4)	0.0002 (4)	−0.0049 (4)
C3	0.0142 (4)	0.0154 (5)	0.0132 (4)	−0.0055 (3)	−0.0019 (3)	−0.0026 (3)
C4	0.0167 (4)	0.0160 (5)	0.0142 (4)	−0.0053 (4)	0.0002 (4)	−0.0042 (4)
C5	0.0197 (5)	0.0193 (5)	0.0135 (4)	−0.0077 (4)	0.0001 (4)	−0.0037 (4)
C6	0.0240 (5)	0.0281 (6)	0.0127 (5)	−0.0088 (4)	0.0012 (4)	0.0001 (4)
C7	0.0281 (6)	0.0148 (5)	0.0211 (5)	−0.0040 (4)	−0.0051 (4)	−0.0001 (4)
C8	0.0165 (5)	0.0172 (5)	0.0147 (4)	−0.0016 (4)	−0.0020 (4)	−0.0012 (4)
C9	0.0128 (4)	0.0148 (4)	0.0130 (4)	−0.0019 (3)	0.0005 (3)	−0.0010 (3)
C10	0.0134 (4)	0.0119 (4)	0.0130 (4)	0.0012 (3)	−0.0006 (3)	−0.0013 (3)
C11	0.0155 (4)	0.0145 (5)	0.0163 (5)	−0.0004 (4)	0.0023 (4)	0.0000 (4)
C12	0.0121 (4)	0.0147 (5)	0.0255 (5)	−0.0011 (3)	0.0002 (4)	−0.0005 (4)
C13	0.0186 (5)	0.0194 (5)	0.0249 (5)	−0.0028 (4)	−0.0066 (4)	−0.0041 (4)
C14	0.0288 (6)	0.0295 (6)	0.0129 (5)	0.0024 (5)	−0.0037 (4)	−0.0043 (4)
Cl1	0.01256 (10)	0.01683 (11)	0.01659 (11)	−0.00351 (8)	0.00141 (8)	−0.00440 (8)
Cl2	0.02090 (12)	0.01512 (11)	0.01719 (11)	−0.00582 (9)	0.00273 (9)	−0.00613 (9)
Cl3	0.01644 (11)	0.01347 (11)	0.01837 (11)	−0.00359 (8)	−0.00078 (9)	0.00147 (8)
Cl4	0.01623 (11)	0.02174 (12)	0.01760 (11)	−0.00576 (9)	−0.00483 (9)	−0.00201 (9)
N1	0.0245 (5)	0.0151 (4)	0.0156 (4)	−0.0066 (3)	−0.0036 (4)	−0.0004 (3)
N2	0.0188 (4)	0.0170 (4)	0.0137 (4)	−0.0056 (3)	−0.0005 (3)	−0.0004 (3)
N3	0.0154 (4)	0.0178 (4)	0.0218 (5)	−0.0020 (3)	−0.0059 (3)	−0.0027 (3)
N4	0.0183 (4)	0.0202 (4)	0.0134 (4)	−0.0017 (3)	−0.0024 (3)	−0.0032 (3)
Zn1	0.01192 (6)	0.01210 (6)	0.01234 (6)	−0.00284 (4)	−0.00084 (4)	−0.00200 (4)

### Geometric parameters (Å, °)

**Table:**

C1—N1	1.3533 (15)	C9—C10	1.4263 (14)
C1—C2	1.3634 (16)	C9—H9	0.9500
C1—H1	0.9500	C10—N4	1.3378 (14)
C2—C3	1.4274 (15)	C10—C11	1.4268 (15)
C2—H2	0.9500	C11—C12	1.3635 (17)
C3—N2	1.3352 (13)	C11—H11	0.9500
C3—C4	1.4266 (14)	C12—N3	1.3476 (15)
C4—C5	1.3627 (15)	C12—H12	0.9500
C4—H4	0.9500	C13—N4	1.4633 (15)
C5—N1	1.3513 (15)	C13—H13A	0.9800
C5—H5	0.9500	C13—H13B	0.9800
C6—N2	1.4658 (14)	C13—H13C	0.9800
C6—H6A	0.9800	C14—N4	1.4631 (15)
C6—H6B	0.9800	C14—H14A	0.9800
C6—H6C	0.9800	C14—H14B	0.9800
C7—N2	1.4641 (15)	C14—H14C	0.9800
C7—H7A	0.9800	Cl1—Zn1	2.2983 (3)
C7—H7B	0.9800	Cl2—Zn1	2.2747 (3)
C7—H7C	0.9800	Cl3—Zn1	2.2831 (3)
C8—N3	1.3530 (14)	Cl4—Zn1	2.2409 (3)
C8—C9	1.3628 (15)	N1—H1A	0.8800
C8—H8	0.9500	N3—H3	0.8800
			
N1—C1—C2	121.51 (10)	C12—C11—C10	119.87 (10)
N1—C1—H1	119.2	C12—C11—H11	120.1
C2—C1—H1	119.2	C10—C11—H11	120.1
C1—C2—C3	119.63 (10)	N3—C12—C11	121.40 (10)
C1—C2—H2	120.2	N3—C12—H12	119.3
C3—C2—H2	120.2	C11—C12—H12	119.3
N2—C3—C4	121.16 (10)	N4—C13—H13A	109.5
N2—C3—C2	122.02 (10)	N4—C13—H13B	109.5
C4—C3—C2	116.81 (9)	H13A—C13—H13B	109.5
C5—C4—C3	120.18 (10)	N4—C13—H13C	109.5
C5—C4—H4	119.9	H13A—C13—H13C	109.5
C3—C4—H4	119.9	H13B—C13—H13C	109.5
N1—C5—C4	121.05 (10)	N4—C14—H14A	109.5
N1—C5—H5	119.5	N4—C14—H14B	109.5
C4—C5—H5	119.5	H14A—C14—H14B	109.5
N2—C6—H6A	109.5	N4—C14—H14C	109.5
N2—C6—H6B	109.5	H14A—C14—H14C	109.5
H6A—C6—H6B	109.5	H14B—C14—H14C	109.5
N2—C6—H6C	109.5	C5—N1—C1	120.78 (10)
H6A—C6—H6C	109.5	C5—N1—H1A	119.6
H6B—C6—H6C	109.5	C1—N1—H1A	119.6
N2—C7—H7A	109.5	C3—N2—C7	120.52 (9)
N2—C7—H7B	109.5	C3—N2—C6	121.19 (10)
H7A—C7—H7B	109.5	C7—N2—C6	118.25 (9)
N2—C7—H7C	109.5	C12—N3—C8	121.00 (10)
H7A—C7—H7C	109.5	C12—N3—H3	119.5
H7B—C7—H7C	109.5	C8—N3—H3	119.5
N3—C8—C9	120.80 (10)	C10—N4—C14	121.26 (10)
N3—C8—H8	119.6	C10—N4—C13	120.79 (9)
C9—C8—H8	119.6	C14—N4—C13	117.89 (10)
C8—C9—C10	120.37 (10)	Cl4—Zn1—Cl2	111.223 (12)
C8—C9—H9	119.8	Cl4—Zn1—Cl3	110.797 (11)
C10—C9—H9	119.8	Cl2—Zn1—Cl3	111.809 (13)
N4—C10—C9	121.08 (10)	Cl4—Zn1—Cl1	117.034 (13)
N4—C10—C11	122.37 (10)	Cl2—Zn1—Cl1	103.260 (10)
C9—C10—C11	116.54 (10)	Cl3—Zn1—Cl1	102.256 (10)
			
N1—C1—C2—C3	−0.32 (17)	C4—C5—N1—C1	−0.92 (17)
C1—C2—C3—N2	178.17 (10)	C2—C1—N1—C5	1.62 (17)
C1—C2—C3—C4	−1.56 (15)	C4—C3—N2—C7	−4.66 (16)
N2—C3—C4—C5	−177.50 (10)	C2—C3—N2—C7	175.62 (10)
C2—C3—C4—C5	2.24 (15)	C4—C3—N2—C6	173.16 (10)
C3—C4—C5—N1	−1.05 (16)	C2—C3—N2—C6	−6.56 (16)
N3—C8—C9—C10	0.00 (16)	C11—C12—N3—C8	0.33 (16)
C8—C9—C10—N4	178.74 (10)	C9—C8—N3—C12	0.33 (16)
C8—C9—C10—C11	−0.90 (15)	C9—C10—N4—C14	176.17 (10)
N4—C10—C11—C12	−178.11 (10)	C11—C10—N4—C14	−4.20 (16)
C9—C10—C11—C12	1.53 (15)	C9—C10—N4—C13	−6.40 (16)
C10—C11—C12—N3	−1.29 (16)	C11—C10—N4—C13	173.22 (10)

### Hydrogen-bond geometry (Å, °)

**Table:**

*D*—H···*A*	*D*—H	H···*A*	*D*···*A*	*D*—H···*A*
N3—H3···Cl1^i^	0.88	2.88	3.5090 (11)	130.
N3—H3···Cl3^i^	0.88	2.46	3.2224 (10)	146.
N1—H1A···Cl2	0.88	2.81	3.4043 (10)	126.
N1—H1A···Cl1	0.88	2.53	3.2066 (10)	134.

Symmetry codes: (i) −*x*, −*y*+1, −*z*.

## References

[bb1] Albrecht, A. S., Landee, C. P. & Turnbull, M. M. (2003). *J. Chem. Crystallogr.* **33**, 269–276.

[bb2] Bruker (2009). *APEX2*, *SAINT* and *SADABS* Bruker AXS Inc, Madison, Wisconsin, USA.

[bb3] El Glaoui, M., Smirani, W., Lefebvre, F., Rzaigui, M. & Ben Nasr, C. (2008). *Can. J. Anal. Sci. Spectrosc.* **25**, 103–107.

[bb4] Huskins, B. F. & Robson, R. (1990). *J. Am. Chem. Soc.* **112**, 1546–1554.

[bb5] Kobel, W. & Hanack, M. (1986). *Inorg. Chem.* **25**, 103–107.

[bb6] Pierpont, C. G. & Jung, O. (1994). *J. Am. Chem. Soc.* **116**, 2229–2230.

[bb7] Sheldrick, G. M. (2008). *Acta Cryst.* A**64**, 112–122. 10.1107/S0108767307043930 18156677

